# Supplementary material for: Melanella martarum sp. nov. (Gastropoda: Eulimidae): the first parasitic deep-sea snail reported for the Salas & Gomez Ridge
Source: PeerJ. 2024 Apr 23;12:e16932. doi: 10.7717/peerj.16932 (PMC11048058; doi:10.7717/peerj.16932)
Supplement: Supplemental Information 2 [file peerj-12-16932-s002.docx]

Supplementary Material

***Melanella martarum* sp. nov. (Gastropoda: Eulimidae): the first parasitic deep-sea snail reported for the Salas & Gomez Ridge.**

Leonardo Santos de Souza^1^, Cynthia M. Asorey^2,3^, Javier Sellanes^2,3^

^1^ Departamento de Zoologia - Instituto de Biociências, Universidade Federal do Rio Grande do Sul (UFRGS), Brazil.
^2^ Centro de Ecología y Manejo Sustentable de Islas Oceánicas (ESMOI), Departamento de Biología Marina, Universidad Católica del Norte, Coquimbo, Chile.

^3^Sala de Colecciones Biológicas, Universidad Católica del Norte, Coquimbo, Chile.

Corresponding Author:
Cynthia M. Asorey
Larrondo 1281, Coquimbo, 1781421, Chile

Email address: cynthia.asorey@ucn.cl


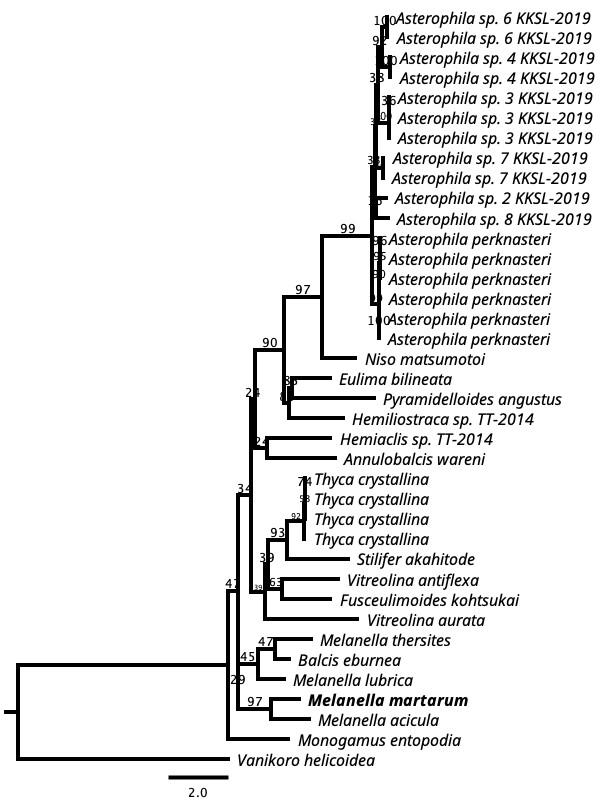


Fig S1: RaxML inferred phylogenetic reconstruction based on 630-bp alignment of partial COI sequences of Eulimidae (bootstrap = 1,000). Bootstrap values obtained are shown in each node. *Vanikoro helicoidea* (Vanikoridae) was used as outgroup.

Table S1: Species used in RaxML inferred phylogenetic reconstruction based on 630-bp alignment of partial COI sequences of Eulimidae with GenBank accession numbers and the authors of each one. Accession numbers of newly obtained sequences are given in bold.

| Family Species | | COI |
| --- | --- | --- |
| *Eulimidae* | *Hemiaclis sp.* TT-2014 | AB930465 |
|  | *Hemiliostraca sp.* TT-2014 | AB930466 |
|  | *Melanella acicula* | AB930464 |
|  | *Monogamus entopodia* | AB930458 |
|  | *Niso matsumotoi* | AB930469 |
|  | *Pyramidelloides angustus* | AB930470 |
|  | *Stilifer akahitode* | AB930461 |
|  | *Thyca crystallina* | AB930460 |
|  | *Vitreolina aurata* | AB930457 |
|  | *Asterophila perknasteri* | MN224306 |
|  | *Asterophila perknasteri* | MN224310 |
|  | *Asterophila sp 4 KKSL-2019* | MN224348 |
|  | *Asterophila perknasteri 3* | MN224362 |
|  | *Fusceulimoides kohtsukai* | LC726231.1 |
|  | ***Melanella martarum* sp. nov.** | **OP577852** |
|  | *Annulobalcis wareni* | JF717841.1 |
|  | *Asterophila perknasteri* | MN224312.1 |
|  | *Asterophila perknasteri* | MN224315.1 |
|  | *Asterophila perknasteri* | MN224316.1 |
|  | *Asterophila perknasteri* | MN224362 |
|  | *Asterophila sp. 2 KKSL-2019* | MN224340.1 |
|  | *Asterophila sp. 3 KKSL-2019* | MN224319. |
|  | *Asterophila sp. 3 KKSL-2019* | MN224324.1 |
|  | *Asterophila sp. 3 KKSL-2019* | MN224325.1 |
|  | *Asterophila sp. 4 KKSL-2019* | *MN224347* |
|  | *Asterophila sp. 6 KKSL-2019* | *MN224311.1* |
|  | *Asterophila sp. 6 KKSL-2019* | *MN224341.1* |
|  | *Asterophila sp. 7 KKSL-2019* | *MN224307.1* |
|  | *Asterophila sp. 7 KKSL-2019* | *MN224336.1* |
|  | *Asterophila sp. 8 KKSL-2019* | *MN224333.1* |
|  | *Balcis eburnea* | *AF120636* |
|  | *Eulima bilineata* | *MG934894* |
|  | *Fusceulimoides kohtsukai* | *LC726231.1* |
|  | *Hemiaclis sp. TT-2014* | *AB930465* |
|  | *Hemiliostraca sp. TT-2014* | *AB930466* |
|  | *Melanella acicula* | *AB930464* |
|  | *Melanella lubrica* | *MG935328.1* |
|  | *Melanella thersites* | *KF644012* |
|  | *Monogamus entopodia* | *AB930458* |
|  | *Niso matsumotoi* | *AB930469* |
|  | *Pyramidelloides angustus* | *AB930470* |
|  | *Stilifer akahitode* | *AB930461* |
|  | *Thyca crystallina* | *AB930460* |
|  | *Thyca crystallina* | *FJ386279.1* |
|  | *Thyca crystallina* | *FJ386280.1* |
|  | *Thyca crystallina* | *FJ386281.1* |
|  | *Vanikoro helicoidea* | *AB930487* |
|  | *Vitreolina antiflexa* | *MT920177* |
|  | *Vitreolina aurata* | *AB930457* |
| *Vanikoridae* | *Vanikoro helicoidea* | AB930487 |
